# Etiological Relation of Autointoxication and Autoinfection to Diseases of the Eye1Read at the meeting of the New York State Medical Association, October 26, 1899.

**Published:** 1900-01

**Authors:** Alvin A. Hubbell

**Affiliations:** Clinical professor of ophthalmology in the University of Buffalo, Buffalo, N. Y.


### BUFFALO MEDICAL JOURNAL.

VOL. XXXIX.—LV. JANUARY, 1900. No. 6.

### Original Communications.

### ETIOLOGICAL RELATION OF AUTOINTOXICATION AND AUTOINFECTION TO DISEASES OF THE EYE.[1]

BY ALVIN A. HUBBELL, M. D.,
Clinical professor of ophthalmology in the University of Buffalo, Buffalo, N. Y.

AUTOINTOXICATION has long been recognised in the history and etiology of disease, but under other names. Netter, in 1884, was the first to use the term, but in a sense somewhat technical and narrow. It was the eminent physician, Bouchard, who gave the term its present signification by broadening its scope so as to include poisoning, not only from materials normally present in the tissues, but from toxic products due to bacterial activity or tissue metabolism.

Autointoxication, then, is self-poisoning, whether by the excess or retention of normal products, or by abnormal products generated within the body. The term should be used in a different sense than that of autoinfection, which implies a direct germ invasion and activity, and is not of as common occurrence. It is possible that a septicemia may come from infection started within the body, or that under extraordinary circumstances, the bacterium coli, so universally present in the intestines, and perhaps normally so, may become active and lead to inflammatory processes near its habitat or even in remote parts of the body. I think I have seen a case of this kind in a man about seventy years of age upon whom I operated for cataract several years ago. I had performed a small iridectomy preliminary to the extraction, which was made about four weeks afterward. Recovery from both operations was rapid and complete. The patient had for years been subject to an obstinate constipation, and being rather

1. Read at the meeting of the New York State Medical Association, October 26, 1899.

careless in his habits and toilet, he would often resort to mechanical measures for securing relief, and the instrument nearest at hand and the most convenient for him was his finger. Before operating, as is always my custom, I took the precaution to free his bowels, for by so doing much infectious and toxic material is eliminated, and there is therefore less danger of inflammatory reaction. This patient was dismissed from the hospital two weeks after the cataract operation, cured. A few weeks later he was fitted to a glass with which vision V.=5-6, nearly normal, and he went home. In about three months he returned, suffering from a severe iridocyclitis with hypopyon. On inquiry I found that he was chronically constipated, and had unduly exposed himself to cold; and it has always been my belief that, through exposure and the nonresisting condition of the parts which this and the operation had brought about, infection took place. This infection was undoubtedly from within, as there was no corneal ulceration or other external communication, and the inflammation was of the iris and ciliary body. I have not been able to rid myself of the idea that this infection also came from the colon bacilli which must have been superabundant in the intestines, and possibly present in other parts of the body, even in the tissues of the eye. I may add that after evacuating the pus by means of a corneal incision, the patient recovered with vision 5-12. A few other experiences have led me to attribute the unusual reactions presented to autoinfection, or perhaps to endoinfection, which I believe to be a term more fully covering the conditions. Infective endocarditis, otitis media in cases of pneumonia or influenza, tubercular arthritis, metastatic orchitis in mumps, etc., are undoubted examples of endoinfection.

Auto- and endoinfection, are therefore not uncommon, but in disease generally they are infrequent as compared with autointoxication. The latter is a wide-spread condition, and may not only accompany the infectious processes, as in typhoid fever, diphtheria, etc., but may originate independently through the development of toxic agencies in the digestive tract and other cavities, and by the metabolism of the tissues of the body. The contents of the gastro-intestinal canal are nutrient-culture media for myriads of bacteria, and the products of their activity are supposed to be very numerous, this activity varying according to the character of the ingesta, the gastro-intestinal secretions and the peristaltic movements. These products may be gases, acids and their compounds, various compound alkalies (indol, phenol, skatol, cresol, tyrosin), and numerous ferments, enzymes, ptomaines and toxalbumins. These may be taken up into the circulation and carried to all parts of the body, and veritable poisonings are the result. Undoubtedly, bacterial action upon the contents of other cavities oftentimes develops toxic products, which may also gain access to the circulation and cause abnormal conditions.

Of no less importance in the production of autointoxication are the normal and abnormal products of tissue-metabolism. Excess of the former, as in over-secretion, diminished excretion or lack of chemical metamorphosis, as illustrated in thyroid disease, in nephritis, and in cirrhosis of the liver, and the presence of the latter, as in glycosuria and other diseases, become equally deleterious.

It is impossible for me to enter into a detailed consideration of the origin and nature of the numerous toxic agencies whose influence are so potent in the development and perpetuation of disease, both of mind and body. Reference must be made to the researches begun by Bouchard and first published by him in 1886 (*Union Médicale*), and continued and extended by many others, both in this country and abroad.

While there is still very much to be desired in regard to determining the exact source and nature of germ-processes, and germ and metabolic products, yet sufficient is known to make their existence and potency undoubted. The recognition of infectious agencies operating both from without and within, and of toxic products both from bacterial processes and metabolic changes, is radically changing the whole aspect of etiology and pathology, and must also bear fruit in the medical therapeutics of the future, as it has already done in the surgical therapeutics of the last two decades.

In diseases of the eye, autointoxication and auto- or endoinfection, have most important bearings upon etiology and treatment. Here, as elsewhere in the body, we must recognise two factors in disease. First, that unknown, but real, something which we call vitality, or vital power, or cell activity, of the parts, the loss of which means atrophy or death, the excess of which means hyperplasia, and the perversion of which means degeneration or neoplasmic growth; second, a *materies morbi*, either chemical or living, in the presence of which cell life ceases, becomes perverted, or is over-active. Cell integrity, cell vigor, vital strength, is the greatest security to health. Whatever impairs this weakens the "defenses," and morbific agencies, otherwise inert, obtain a foothold. This cell vigor depends upon conditions of inheritance and of cell nutrition. The former are transmitted from parent to child, and the latter are influenced by a multitude of circumstances of nerve reactions, supply of food and oxygen, free excretion, muscular activities and rest. In the former there is "robustness" of tissue and absence of disease. In the latter there are dyscrasias and an ever-present "morbid opportunity," in which morbific influences are not repelled and disease follows.

Thus the eye, with the body generally, may possess an inherent "vital strength" which defies even extraordinary morbific agencies; or, on the other hand, it may scarcely have vital resources sufficient to resist the slightest extraneous influence. In considering diseases of the eye, then, etiologically or therapeutically, this question of tissue robustness or vital resistance, determined mainly by studying that of other parts of the body, is of greatest importance; for in no other way can we understand why the eyes of one person readily yield to endoinfection or to endogenetically derived poisons, while in those of another no effect whatever is apparent.

Among the diseases of the eye which can be clearly recognised as auto- or endoinfectious, are those due to syphilis, both acquired and congenital. What the infective material of syphilis is, has not yet been determined, but there is no reason to doubt its bacterial character. Whether secondary and tertiary manifestations of syphilis are directly due to the same germ as the primary ones, or whether they arise from some peculiar toxins derived from the activities of the supposed bacterial germs in the primary disease cannot yet be determined. But whatever be the agent, the eye, under suitable conditions of vital nonresistance, becomes a favorite soil for its operations. Affections of the eye are seen in the secondary stages of syphilis in the form of acute inflammation, especially of the iris, ciliary body and choroid, and sometimes, but usually later, of the retina, cornea and sclera. In the tertiary stages there are chronic inflammations of the choroid, retina and other tissues of the ball, and gummatous infiltrations of the iris and other structures, and also atrophies and paralyses of the optic and motor nerves, found in connection with cerebral and spinal sclerosis. In inherited syphilis, it is not possible that syphilitic germs, if they exist, can be transmitted from parent to offspring; but a lowered tissue vitality is transmitted, and the eye structures are easily affected by certain toxins and infections, whereby interstitial keratitis, and chronic inflammation of the inner structures of the eye frequently develop.

Tuberculosis is another endoinfectious disease of the eye, and may attack any part; but it is seen most frequently in the choroid. The agent of infection is well known, and the process well illustrates what may happen, in a modified form, in other endoinfections of the eye, such as those arising from influenza, leprosy, variola, measles, and the like. Septic endoinfection of the eye may occur in pyemia, the septic fevers and so forth, causing purulent inflammations and even panophthalmitis. Many other illustrations of undoubted ocular endoinfection might be cited, but the above are sufficient for the present purpose.

Let us now for a moment, turn our attention to some of the autointoxications, or those diseases in which there is evident poisoning by reason of the excess of some normal tissue-product, or the presence of some abnormal substance generated somewhere within the body. Uricacidemia is perhaps a pertinent illustration of an excess of a normal product. The accumulation of uric acid in the blood in abnormal quantity is believed by Haig, of England, and his adherents to be the cause of many of the ills to which human flesh is heir, and the eye does not escape its poisonous influence. An excess of the secretion of the thyroid gland, however this may occur, is now thought by many to be the essential factor in the production of that group of symptoms found in exophthalmic goitre, including the disturbances of vision, the protrusion of the eyeball, retraction of the upper lid, and the impairment of the winking reflex. In acromegaly, the question has been raised as to whether or not the hypertrophy of the pituitary body of the brain does not, in some way, influence some unknown secretory function by which certain bodily constituents are increased, and the peculiar physical abnormalities of the disease developed.

Other diseases are supposed to be caused by normal products, which are present in excess, because they are not chemically neutralised or disintegrated, as in Addison's disease, in which the adrenals have seemed to lose their function, or as in glycosuria; or because they are not sufficiently eliminated, as in nephritis, both acute and chronic, and diseases of the liver, etc. Abnormal products are formed in the body in many ways. But those which arise from the fermenting and putrifying changes common in the alimentary tract, and which develop in the course of infectious processes are of special significance. The intestinal ptomaines and toxalbumins are now believed to be a prolific source of very many nervous and psychic disturbances. In tetanus, it is well-established that a most virulent poison is generated, and it has been estimated that 1-350 of a grain is sufficient to kill a man. It is undoubtedly a product of this kind which causes those manifold sequelæ of diphtheria, including the ocular paralyses. In typhoid fever another poison is evolved which, presumably, is the direct cause of the delirium and many other symptoms, and sometimes, also, of affections of the eyes. The same may be said, with confidence, of pneumonia and its casual infection. Rheumatism and gout are believed to be manifestations of autointoxication, either from some retained metabolic product or from some abnormally derived substance, or perhaps both.

To revert now more specifically to the eye itself, I assert that scarcely a disease of this organ, at least of an inflammatory character, can be named in which the cause, when not due to mechanical injury or to direct, exoinfection, does not lie either in autointoxication or endo- (auto-) infection. Take for example, iritis. The infection of syphilis, or the toxic agent of rheumatism and gout account for it in most cases. In other cases in which it seems to occur spontaneously the vital resistance of the iris may have been lowered by exposure to cold or by some dyscrasia, and then some toxin in the blood may determine an inflammation. Tenontitis is purely akin to rheumatism, and is due to the same morbific agent. The choroid, in its vascularity, is exposed to the same toxic and infective invasions as the iris and choroiditis is a frequent disease. Retinitis may always be regarded as due to some poison in the blood, whether it be from albuminuria, diabetes, syphilis, leukemia, pernicious anemia or arrested menstruation. Again, optic neuritis, both in its papillary and retro-bulbar forms, is distinctly a toxic affection. The most common form of optic neuritis is that seen in connection with intracranial growths and inflammations. There is but one theory which to my mind accounts for this disease, and that is, that a toxin is developed during the progress of an intracranial growth or a cerebral inflammation which is carried to the nerve through the various spaces and lymph-channels related to it, and causes the inflammatory reaction.

It is well known that nicotine and alcohol often produce retro-bulbar optic neuritis; and is it to be presumed that, when these are not operative, it may be caused by other toxic principles, the identity of which future researches will reveal?

Finally, I believe, with Professor Panas, of Paris, that sympathetic ophthalmia furnishes another illustration of autoinfection or auto-intoxication, not in the sense that it is a migratory disease in which germs are transported from the injured to the sound eye, as Deutschmann vainly endeavored to prove experimentally, but in the sense that the uveal tract of the sound eye, by reason of a lowering of its vital tone through the extreme reflex and vaso-motor disturbances kept up by a foreign body in, or injury to, the other eye, becomes truly poisoned or infected by materials in the general circulation to the extent of inducing a most obstinate and uncontrollable inflammation. More than half a century ago, that great pioneer in ophthalmic research, Mackenzie, of Glasgow, advanced the reflex theory of sympathetic ophthalmia. But it has hardly been deemed sufficient to account for all the phenomena of the disease. But to this, add autoinfection of the parts whose vital defenses have been withdrawn by the reflex disturbance, and we have, it seems to me, a full and rational explanation of the cause of this disease.

Without extending illustrations further, I will conclude by adding that while we are but on the threshold of an understanding of autointoxication and autoinfection, yet I believe that we have advanced far enough to gain a glimpse of their verities and their import in the prevention and treatment of disease. By determining their origin and nature means will be found by which to avoid them or to destroy them, and the causes of very many diseases will thus be removed. In therapeutics, also, I believe much will be accomplished when we learn how to eliminate or antidote these poisons.

Admitting the infectious and toxic origin of a large range of diseases, perhaps a large majority, from which no organ of the body would seem to be exempt, the ophthalmologist, otologist, neurologist, gynecologist, surgeon and physician are all, alike, relegated to one common field of study and research and with the one common purpose of seeking and removing or making inoperative the causes of disease.

212 Franklin Street.

